# Two opposite effects of desmoglein 3 on the growth of oral squamous cell carcinoma between anchorage -dependent and -independent conditions

**DOI:** 10.1371/journal.pone.0358339

**Published:** 2026-09-25

**Authors:** Junichirou Inada, Masaki Minabe, Yurie Akiyama, Kazunari Higa, Takeshi Nomura, Michiyoshi Kouno

**Affiliations:** 1 Department of Oral Oncology, Oral and Maxillofacial Surgery, Tokyo Dental College, Chiba, Japan; 2 Department of Dentistry and Oral Surgery, Tokyo Metropolitan Bokutoh Hospital, Tokyo, Japan; 3 Cornea Center Eye bank, Tokyo Dental College Ichikawa General Hospital, Chiba, Japan; 4 Department of Dermatology, Tokyo Dental College Ichikawa General Hospital, Chiba, Japan; Shantou University Medical College, CHINA

## Abstract

Desmoglein 3 (Dsg3), a desmosomal cadherin, is aberrantly expressed in oral squamous cell carcinoma (OSCC), although its role remains controversial. Higher Dsg3 expression has been reported in metastatic lymph nodes compared with primary tumors. This study aimed to clarify the role of Dsg3 under anchorage-dependent (AD) and anchorage-independent (AID) conditions. Cell lines derived from primary tumors (P) and metastatic lymph nodes (LY) of three OSCC patients, along with two commercial OSCC cell lines, were analyzed for proliferation, migration, and multicellular aggregate (MCA) formation. Differentially expressed genes (DEGs) following Dsg3 knockdown were also examined, followed by Gene Ontology (GO) analysis. Under AD conditions, Dsg3-low cells showed significantly higher proliferation and migration than Dsg3-high cells (p < 0.05). In contrast, under AID conditions, Dsg3 expression was markedly upregulated (up to 50-fold), and Dsg3-high cells exhibited enhanced proliferation. However, Dsg3 knockdown significantly increased proliferation under AD conditions but had no significant effect under AID conditions. Instead, under AID conditions, Dsg3 knockdown significantly reduced aggregate size and increased variability in circularity, indicating impaired structural organization of MCAs. Consistent with these findings, DEG analysis revealed no enrichment of apoptosis- or cell cycle–related pathways under AID conditions. GO analysis indicated that DEGs under AD conditions were associated with macromolecule biosynthesis, whereas those under AID conditions were related to gene expression and nucleic acid biosynthesis. These results indicate that Dsg3 exerts anchorage-dependent functions in OSCC, suppressing proliferation under AD conditions while contributing to the structural organization of multicellular aggregates under AID conditions. This context-dependent role may underlie the paradoxical effects of Dsg3 and facilitate tumor adaptation to metastatic environments.

## 1. Introduction

Desmoglein 3 (Dsg3), the target antigen of pemphigus vulgaris, is a key component of keratinocyte cell-cell adhesion. Recent studies have reported that Dsg3 is overexpressed in head and neck squamous cell carcinoma (HNSCC) [1], where it promotes cancer progression through intercellular signal transduction [2,3]. High Dsg3 expression in lymph node micrometastases of HNSCC suggests its potential utility as a predictive biomarker for lymph node metastasis [4]. These findings indicate that Dsg3 overexpression contributes to tumor progression and lymph node metastasis.

Previously, we demonstrated that metastatic lymph node cells exhibit significantly higher DSG3 expression compared to primary tumor cells from the same OSCC patients [5]. Generally, cancer cells possess the ability to grow under anchorage-independent (AID) conditions and establish metastatic tumors by forming colonies [6–8]. Overexpression of Dsg2, another desmosomal cadherin isoform, has been shown to deregulate multiple signaling pathways associated with AID cell survival [9]. These findings suggest that Dsg3 overexpression may be critical for OSCC cell growth in scaffold-less metastatic lymph nodes.

Conversely, other studies have reported that Dsg3 downregulation is involved in tumor progression. Dsg3 expression is reduced in OSCC tissues compared to normal tissues, particularly at the invasion front, and is associated with aggressive clinicopathological features [10–13]. This reduction in desmosomal cell-cell adhesion facilitates the invasion and metastasis of OSCC cells following malignant transformation.

These paradoxical effects of Dsg3 on tumor progression suggest that the role of desmosomal cadherins in cancer promotion is multifaceted and complex. Recent pathological studies have shown that desmosomal protein expression varies by cancer stage, increasing in hyperplastic tissues while decreasing in dysplastic and tumor tissue [14]. In vitro studies have further indicated that cancer progression may depend on Dsg3 mutations and the culture environment [15]. Based on these findings, we hypothesize that the effects of Dsg3 expression on cancer cell growth are influenced by the anchorage environment. Therefore, this study investigates the role of Dsg3 expression in OSCC growth under anchorage-dependent (AD) and anchorage-independent (AID) conditions.

## 2. Materials and methods

### 2-1. Cell lines and cell cultures

The TOSCa series, consisting of six OSCC cell lines, was established from the primary tumors (P) and metastatic lymph nodes (LY) of three OSCC patients who underwent surgery at the Showa University Dental Hospital, Japan over a period of approximately five years, from August 15, 2002, to August 14, 2007 [5,16]. Specifically, cell lines derived from primary tumors and metastatic lymph nodes of the same patients were designated as P and LY, respectively (Table 1). Two commercial human OSCC cell lines (HSC3 and SAS) were purchased from the Japanese Cancer Research Resources Bank (JCRB). Cell lines were maintained in Dulbecco’s Modified Eagle’s Medium/Nutrient Mixture F-12 Ham with L-glutamine (DMEM/F12) (Sigma-Aldrich, St. Louis, MO), supplemented with 10% fetal bovine serum (FBS) (Thermo Fisher Scientific, Waltham, MA) and 1% penicillin/streptomycin (Thermo Fisher Scientific). Cells were cultured at 37°C in a humidified atmosphere with 5% CO2.

**Table 1:** Profile of cell lines established from the primary tumor and metastatic lymph nodes of 3 OSCC patients.

Cell line name	Gender	Primary site	Primary tumor	Metastatic lymph node
TOSCa-7	Male	Gingiva	7P	7LY
TOSCa-17	Female	Gingiva	17P	17LY
TOSCa-58	Male	Tongue	58P	58LY

OSCC cells were cultured under two conditions: anchorage-dependent and anchorage-independent. For anchorage-dependent culture, Nunc EasYDish 100 mm plates (Thermo Fisher Scientific) with enhanced hydrophilicity on polystyrene surfaces, but lacking extracellular matrix components, were used. For anchorage-independent culture, EZ-BindShut II six-well plates (IWAKI), coated with 2-methacryloyloxyethyl phosphorylcholine polymer, were employed. Under AD conditions, cells were grown in 100 mm Nunc EasYDish plates (Thermo Fisher Scientific). For AID conditions, cells were maintained in EZ-BindShut II six-well plates (IWAKI).

### 2-2. Dsg3 Knockdown by siRNA

Dsg3 knockdown was performed using Silencer® Select pre-designed siRNA (s4327) (Thermo Fisher Scientific). A non-targeting siRNA (Silencer Select Negative Control #1, Cat #4390843; Thermo Fisher Scientific) was used as a negative control to exclude non-specific effects of siRNA transfection. To minimize off-target effects, we confirmed that similar results were obtained using at least two different siRNA sequences (s4327 and s4329). A 10 µM siRNA solution was transfected into cells using Lipofectamine RNAiMAX (Thermo Fisher Scientific) according to the manufacturer’s protocol. Forty-eight hours after transfection, total RNA was extracted, and Dsg3 knockdown efficiency was confirmed by real-time qPCR.

### 2-3. RNA Extraction and Real-Time RT-PCR

Total RNA was extracted from OSCC cell lines using the RNeasy Micro Kit (Qiagen, Hilden, Germany). First-strand cDNA synthesis was performed using the PrimeScript RT Reagent Kit with gDNA Eraser (Takara, Shiga, Japan) following the manufacturer’s instructions. Real-time qPCR was conducted on an ABI 7000 thermocycler (Thermo Fisher Scientific) using TB Green® Premix Ex Taq™ II (Tli RNaseH Plus). The following primers were used:

- Dsg3 Forward Primer: 5′-CCTGTGCAGCAGCCTGGTAA-3′
- Dsg3 Reverse Primer: 5′-CTCATGCATAAGCAGAGGCACAA-3′
- GAPDH Forward Primer: 5′-GCACCGTCAAGGCTGAGAAC-3′
- GAPDH Reverse Primer: 5′-TGGTGAAGACGCCAGTGGA-3′

Relative gene expression levels were normalized to GAPDH signals. All analyses were performed in triplicate.

### 2-4. Cell proliferation assay

Cell proliferation was analyzed using the Premix WST-1 Cell Proliferation Assay System (Takara). OSCC cells (1 × 10^5^ cells/well) were cultured in 96-well plates for 48 hours. Afterward, 10 µL of WST-1 reagent was added to each well. Following a 2-hour incubation, absorbance at 450 nm was measured using a microplate reader (Bio-Rad Laboratories) to determine cell viability. Proliferation after Dsg3 knockdown was assessed using the same protocol. All assays were performed in triplicate.

### 2-5. Wound healing assay

OSCC cell lines were cultured in six-well plates for 48 hours. After reaching confluency, a scratch was made using a pipette tip. Wound closure was photographed every 2 hours, and the wound area was analyzed after 8 hours using ImageJ software.

### 2-6. Multicellular aggregate size analysis under AID conditions

Multicellular aggregate (MCA) formation was evaluated under AID conditions following Dsg3 knockdown. OSCC cell lines (7LY and HSC3) were seeded in EZ-BindShut®Ⅱ 96-well U-bottom plates at a density of 3 × 10³ cells/100 µL per well and cultured for 48 hours. Aggregates were observed under phase-contrast microscopy, and images were acquired under identical conditions. The projected area and circularity of aggregates were measured using ImageJ software. Aggregate size was quantified as projected area (µm²). All experiments were performed in triplicate.

### 2-7. RNA-sequencing

Total RNA was extracted from OSCC cell lines using the RNeasy Micro Kit (Qiagen) and quantified with a Nanodrop spectrophotometer. RNA integrity was assessed using an Agilent TapeStation. cDNA libraries were prepared using the SMART-Seq® v4 Ultra® Low Input RNA Kit (Takara Bio) and the Nextera XT DNA Library Preparation Kit (Illumina) according to the manufacturers’ protocols. Sequencing was performed on the Illumina NovaSeq 6000 platform. The RNA-seq data have been deposited in the Gene Expression Omnibus (GEO) database under accession number GSE275776.

### 2-8. Identification of Differentially Expressed Genes (DEGs) and Gene Ontology (GO) analysis

Reads shorter than 16 bases were excluded from the analysis. Because of the limited number of samples, statistical significance alone was not used as the criterion for DEG selection, and the RNA-seq analysis was therefore conducted as an exploratory analysis to identify candidate genes associated with Dsg3 knockdown. Candidate differentially expressed genes (DEGs) were identified using a log_2_ fold change threshold (≥1 or ≤−1). After Dsg3 knockdown, candidate DEGs common to Dsg3-positive cells (7LY, SAS) were extracted, and their overlap was visualized with a Venn diagram. GO analysis of the candidate DEGs was conducted using the DAVID web tool (v2022q2) to examine biological processes (BP FAT).

### 2-9. Statistical analysis

Statistical analyses were performed using Student’s t-test or the Mann–Whitney U test for comparisons between two groups. Formal normality testing was not performed because of the limited number of observations. Statistical tests were selected according to the characteristics of each dataset. All experiments were performed at least three independent times, and data are presented as the mean ± standard deviation (SD). Statistical analyses were performed using EZR, and P < 0.05 was considered statistically significant.

## 3. Results

### 3−1. Comparative Analysis of Cell Proliferation and Migration Between Dsg3 Low- and High-Expressing Cells Under AD Conditions

**Fig 1:**
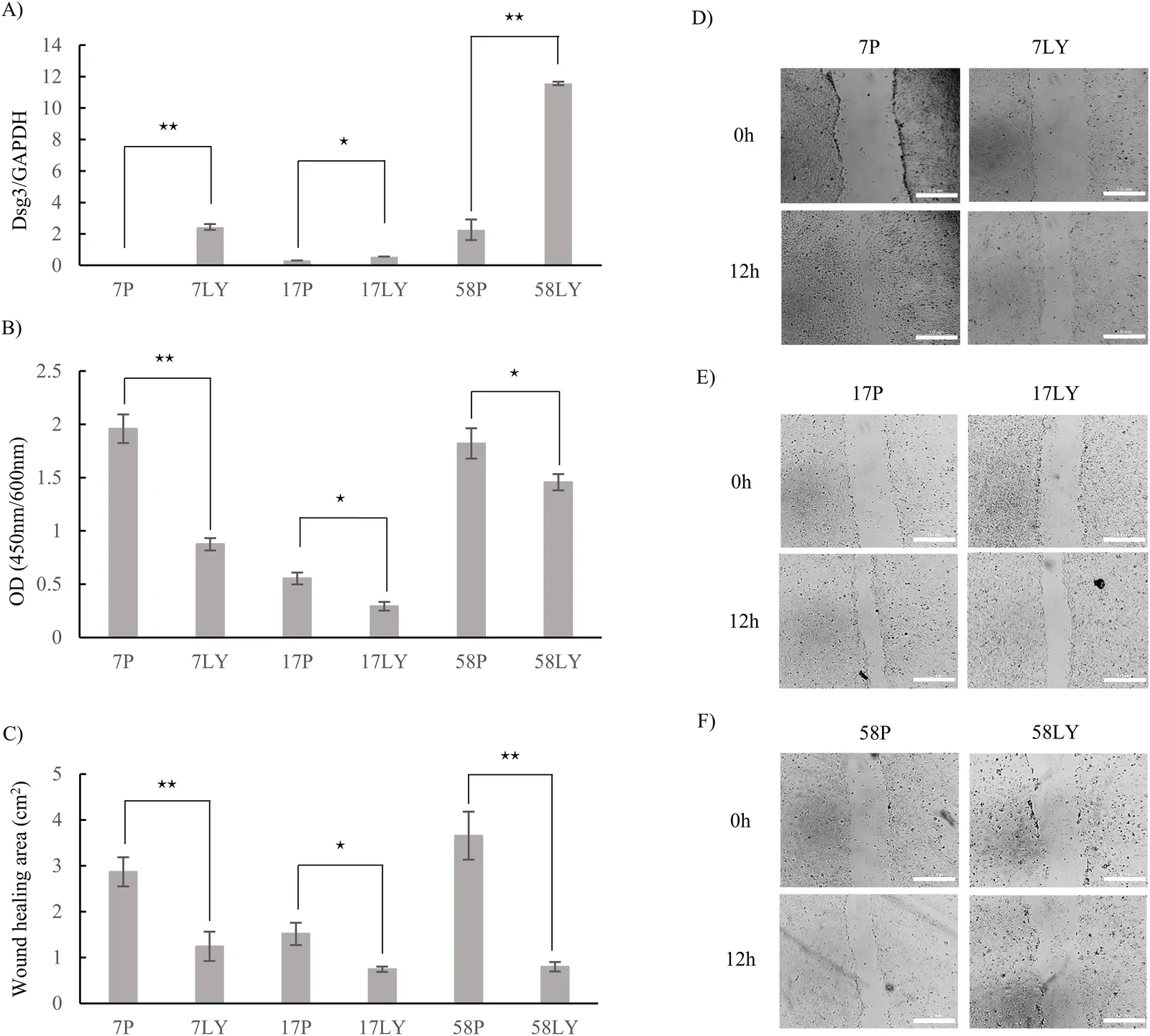
Analysis of the relationship between Dsg3 expression and cell proliferation and migration ability in OSCC cells under AD conditions. (A) Expression of Dsg3 in 6 OSCC cell lines established from the primary tumor and metastatic lymph nodes of 3 OSCC patients. Dsg3 expression was higher in metastatic lymph nodes (LY) than in primary tumor (P). (B) Cell proliferation ability of Dsg3 low-expressing cells was higher than that of Dsg3 high-expressing cells of the same patient under AD conditions. (C) Cell migration ability of Dsg3 low-expressing cells was higher than that of Dsg3 high-expressing cells of the same patient under AD conditions. Statistical analysis was performed using Student’s t-test. Results are presented as mean ± S.D. from three independent experiments. *P < 0.05, **P < 0.01. (D) Photograph of wound closure recorded at 0 hours and 12 hours after wound formation. Wound healing ability of Dsg3 negative cells (7P) has higher than that of Dsg3 positive cells (7LY). Wound closure in 7P was observed at 12h after scratching. (E, F) Wound healing ability of Dsg3 low cells (17P, 58P) was higher than that of Dsg3 high cells (17LY, 58LY). (Scale bar = 1.0 mm).

In this in vitro study, we defined an adhesive culture lacking extracellular matrix components as anchorage-dependent (AD) conditions, while a suspension culture with low-adhesion treatment as anchorage-independent (AID) conditions. We first examined the relationship between Dsg3 expression and cell proliferation and migration under AD conditions. WST-1 cell proliferation assays and migration analyses revealed that cell proliferation and migration abilities were significantly higher in Dsg3-low cells (7P, 17P, 58P) compared to Dsg3-high cells (7LY, 17LY, 58LY) (Figs 1A-1C).

Among all cell lines, the Dsg3-negative cells (7P) exhibited the highest proliferation and migration potential. For example, Dsg3-negative cells (7P) demonstrated nearly twice the growth rate of Dsg3-positive cells from the same patient (7LY). Similarly, 17P (Dsg3-low) showed a 2-fold increase in proliferation and migration compared to 17LY (Dsg3-high), while 58P (Dsg3-low) exhibited a 1.3-fold increase over 58LY (Dsg3-high). In wound closure assays, Dsg3-low cells (7P, 17P, 58P) demonstrated faster wound healing compared to Dsg3-high cells (7LY, 17LY, 58LY) (Figs 1D-1F). Among these, the Dsg3-negative cells (7P) exhibited the fastest wound closure (Fig 1D).

### 3−2. Effect of AID conditions on Dsg3 expression in OSCC cells

**Fig 2:**
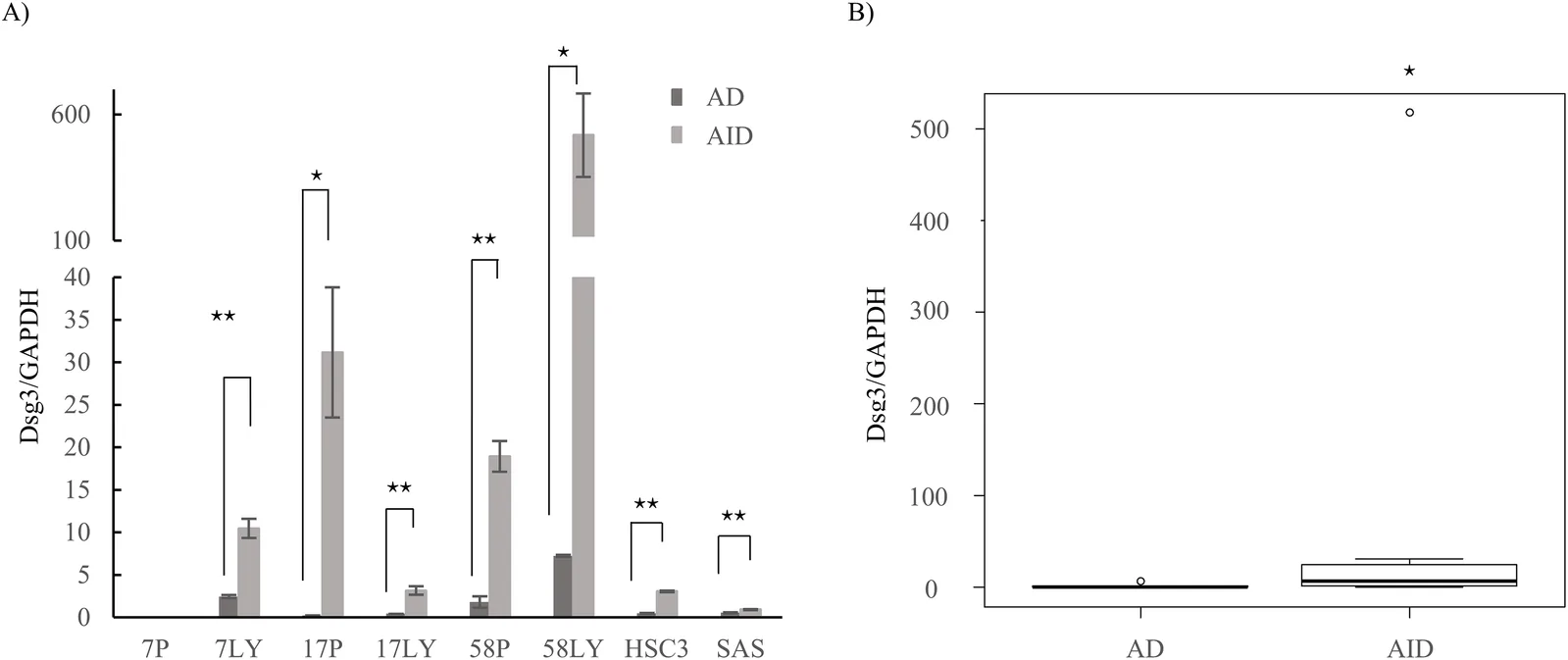
Comparison of Dsg3 expression in OSCC cell lines under AID and AD conditions. (A) Relative Dsg3 mRNA expression normalized to GAPDH under AD and AID conditions in OSCC cell lines. The vertical axis indicates relative expression levels, and the horizontal axis indicates cell lines. Statistical analysis was performed using Student’s t-test. Results are presented as mean ± S.D. *P < 0.05, **P < 0.01. (B) Box-and-whisker plots showing relative Dsg3 expression levels under AD and AID conditions. Statistical analysis was performed using the Mann–Whitney U test. *P < 0.05.

When Dsg3 mRNA expression was analyzed under AID conditions, a significant increase in Dsg3 expression was observed in all cell lines except for the Dsg3-negative cells (7P), compared to AD conditions (Figs 2A, 2B). The other cell lines exhibited an average 28-fold upregulation in Dsg3 expression under AID conditions. In contrast, Dsg3 expression in 7P remained unchanged after transitioning to AID conditions.

### 3-3. Comparative analysis of cell proliferation between Dsg3 low- and high-expressing cells under AID conditions

**Fig 3:**
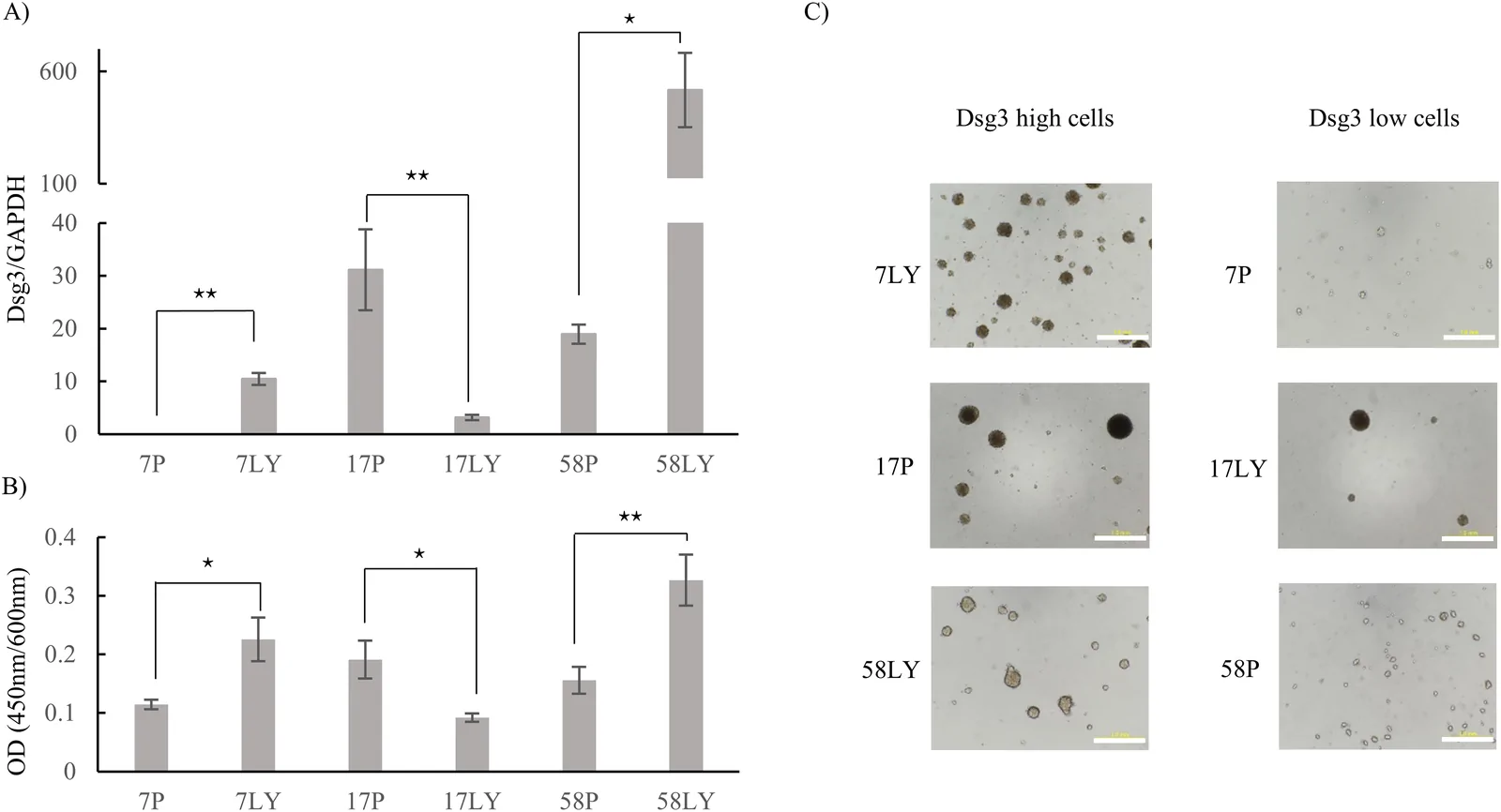
Analysis of cell proliferation under AID conditions. (A, B) Cell proliferation of OSCC cell lines under AID conditions measured by WST-1 assay. The vertical axis represents absorbance at 450 nm, reflecting cell proliferation, and the horizontal axis indicates the cell lines. Statistical analysis was performed using Student’s t-test. Results are presented as mean ± S.D. from three independent experiments. *P < 0.05, **P < 0.01. (C) Representative images of colony formation after 48 hours of incubation. Compared with Dsg3-low cells (7P, 17LY, 58P), Dsg3-high cells (7LY, 17P, 58LY) formed larger colonies. (Scale bar = 1.0 mm).

Next, we investigated the relationship between Dsg3 expression and cell proliferation under AID conditions. Dsg3-high cells (7LY, 17P, 58LY) exhibited significantly greater proliferation than their Dsg3-low counterparts (7P, 17LY, 58P) (Figs 3A, 3B). Among all cell lines, 58LY, which had the highest Dsg3 expression, demonstrated the strongest proliferation under AID conditions. In colony formation assays, Dsg3-high cells (7LY, 17P, 58LY) produced larger colonies compared to Dsg3-low cells (7P, 17LY, 58P) (Fig 3C). Notably, Dsg3-negative cells (7P) exhibited smaller and fewer colonies than all Dsg3-positive cell lines.

### 3-4. Effect of Dsg3 knockdown on cell proliferation under AD and AID conditions

**Fig 4:**
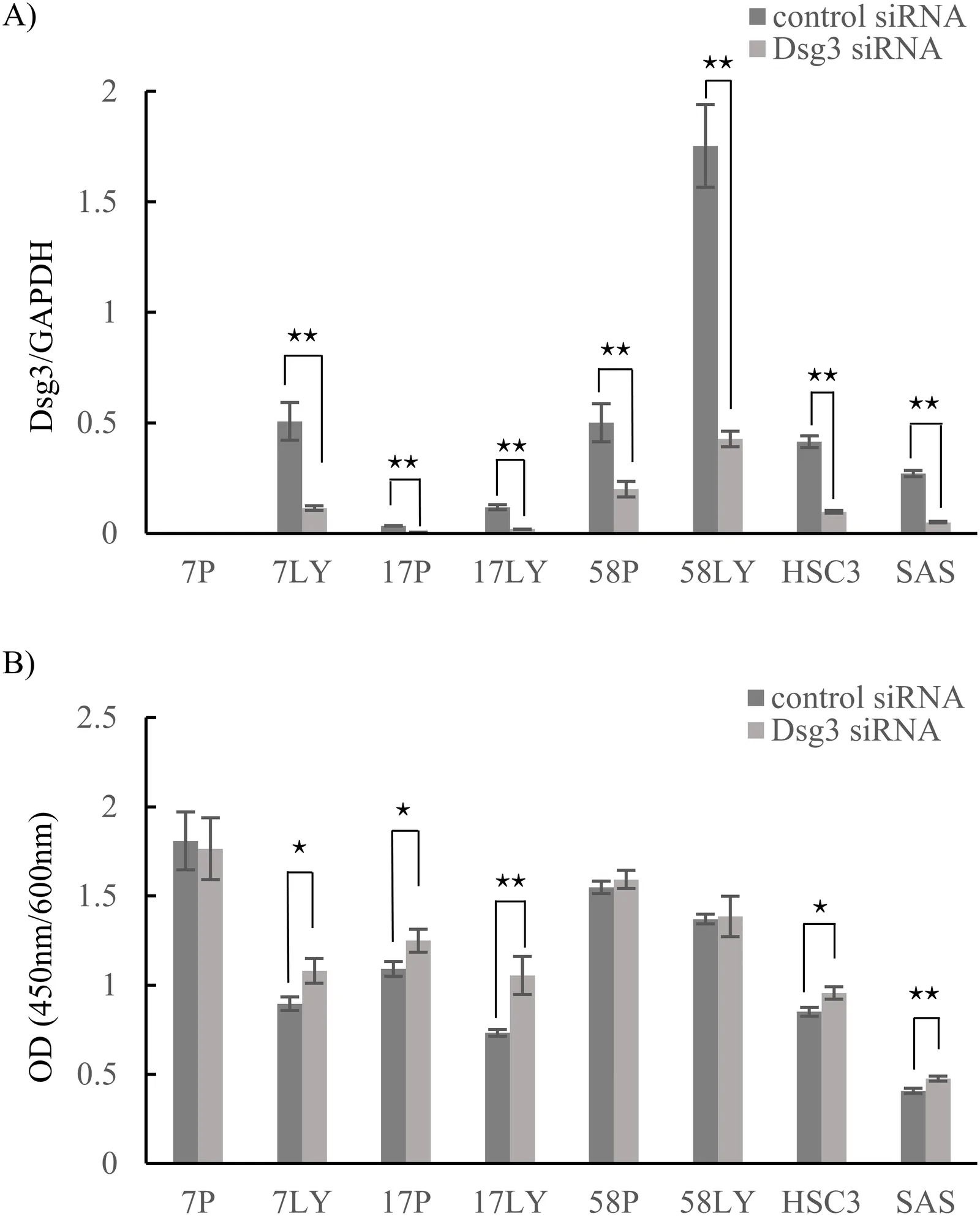
Analysis of cell proliferation ability under AD conditions after Dsg3 knockdown. (A) Dsg3 mRNA expression 48h after Dsg3 knockdown in 6 OSCC cell lines, HSC3 and SAS. (B) Cell proliferation ability after Dsg3 knockdown under AD conditions. Five of 7 Dsg3-positive cell lines showed significant increase of cell proliferation ability. Statistical analysis was performed using Student’s t-test. Results are presented as mean ± S.D. from three independent experiments. *P < 0.05, **P < 0.01.

We next examined the effect of Dsg3 knockdown on cell proliferation under both AD and AID conditions. Dsg3 knockdown significantly reduced Dsg3 mRNA expression in all cell lines except for the Dsg3-negative cells (7P) (Fig 4A). Under AD conditions, Dsg3 knockdown significantly increased cell proliferation in five of seven Dsg3-expressing cell lines (Fig 4B). Although the proliferation of 58P and 58LY also increased, the changes were not statistically significant. Under AID conditions, Dsg3 knockdown caused no significant change in cell proliferation across any cell line.

### 3-5. Effect of Dsg3 Knockdown on Multicellular Aggregate Formation Under AID conditions

**Fig 5:**
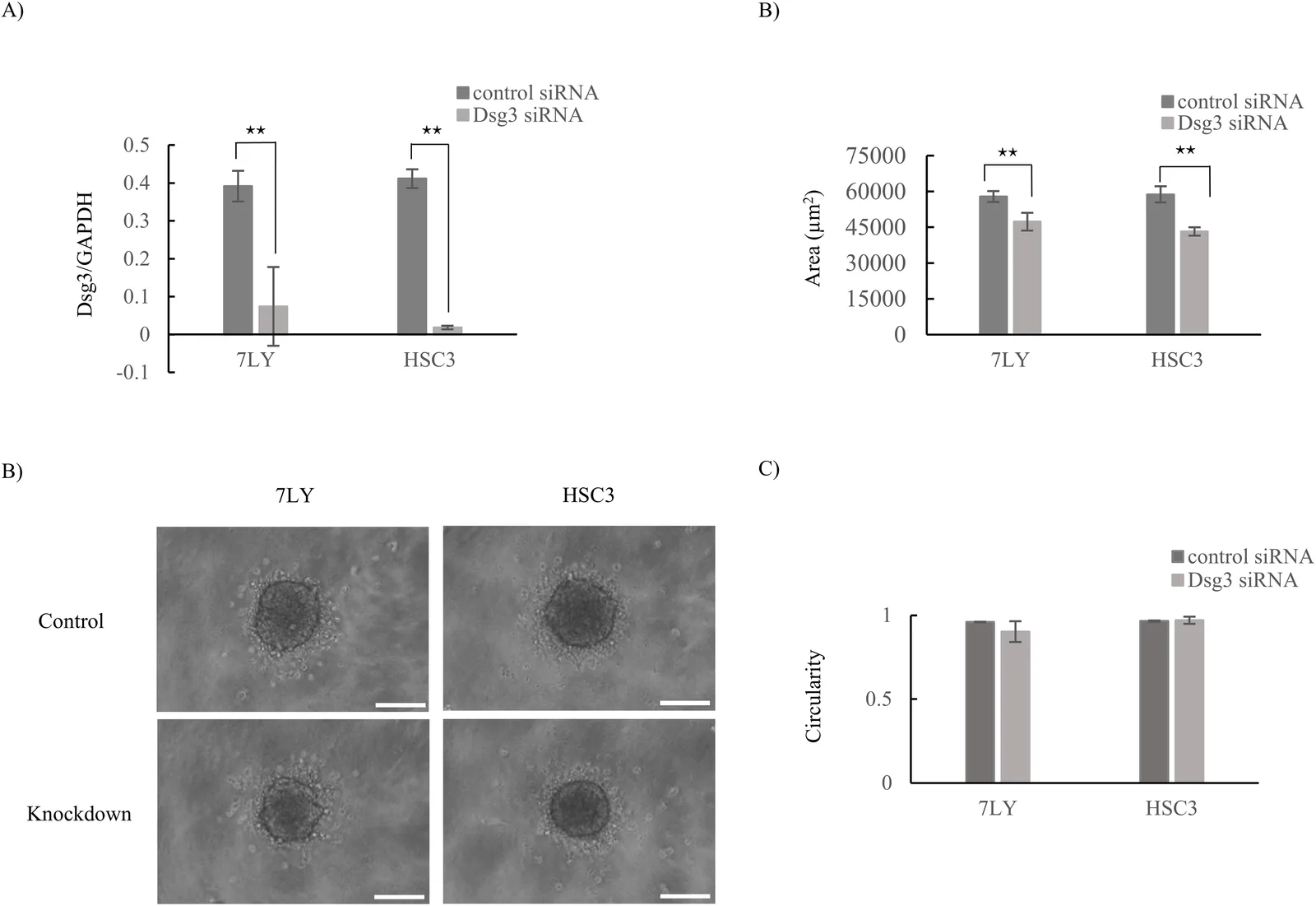
Analysis of MCA size under AID conditions after Dsg3 knockdown. (A) Dsg3 mRNA expression 48h after Dsg3 knockdown in OSCC cell lines.(B) Representative images of multicellular aggregates formed under AID conditions after Dsg3 knockdown. (Scale bar = 200 µm)(C) Quantification of aggregate size measured as projected area (µm²) using ImageJ software. Dsg3 knockdown significantly reduced aggregate size compared with control cells.(D) Circularity analysis of MCA. No significant difference in circularity was observed between control and Dsg3 knockdown groups.Statistical analysis was performed using Student’s t-test. Results are presented as mean ± S.D. from three independent experiments. *P < 0.05, **P < 0.01.

We next examined the effect of Dsg3 knockdown on multicellular aggregate (MCA) formation under AID conditions. Dsg3 knockdown significantly reduced Dsg3 mRNA expression in OSCC cell lines (Fig 5A). Under AID conditions, Dsg3 knockdown significantly reduced aggregate size in both 7LY and HSC3 cells (Fig 5B, 5C). In contrast, no significant change in circularity was observed between control and Dsg3 knockdown groups (Fig 5D).

### 3-6. Gene expression changes associated with Dsg3 knockdown under different anchorage conditions

Finally, to preliminarily explore the molecular mechanisms associated with Dsg3 expression under AD and AID conditions, we performed RNA sequencing analysis. We identified candidate differentially expressed genes (candidate DEGs) following Dsg3 knockdown and extracted those commonly altered in both the patient-derived cell line (7LY) and the commercial cell line (SAS), followed by Gene Ontology (GO) analysis.

**Fig 6:**
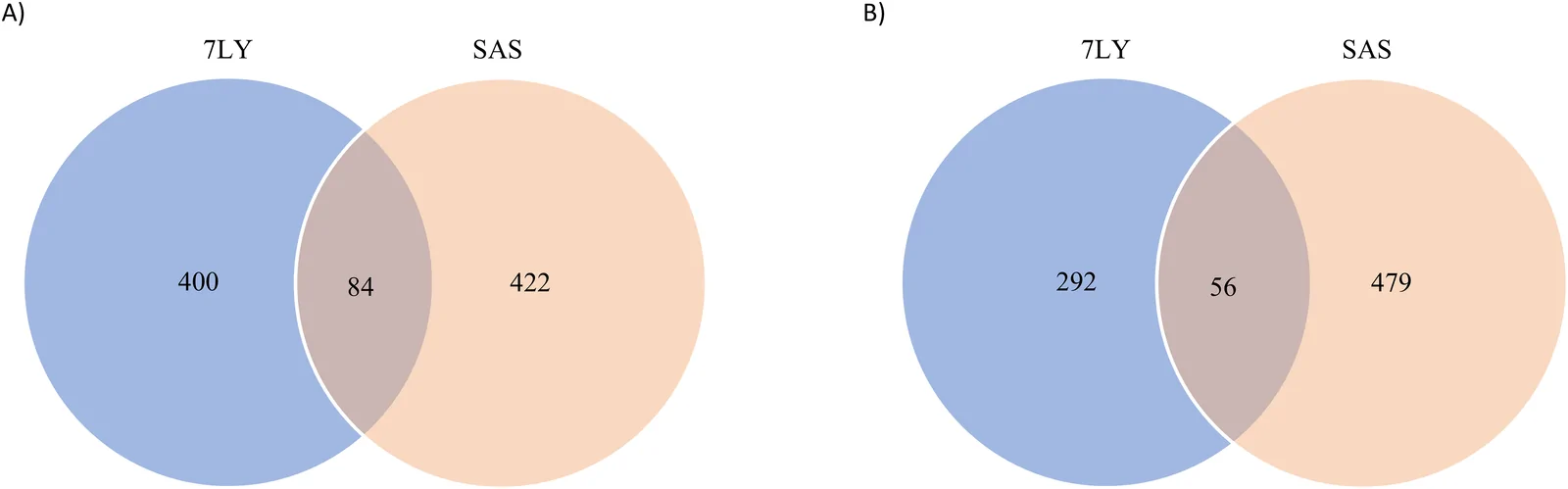
Venn diagrams showing overlapping candidate DEGs following Dsg3 knockdown in Dsg3-positive OSCC cell lines (7LY and SAS). (A) Venn diagram showing overlapping candidate DEGs under AD conditions. Eighty-four overlapping candidate DEGs were identified in Dsg3-positive OSCC cell lines (7LY and SAS). (B) Venn diagram showing overlapping candidate DEGs under AID conditions. Fifty-six overlapping candidate DEGs were identified in Dsg3-positive OSCC cell lines (7LY and SAS).

Under AD conditions, a total of 84 overlapping candidate DEGs were identified in 7LY and SAS cells. Among these, 30 genes were concordantly upregulated and 19 genes were concordantly downregulated in both cell lines (Fig 6A; Tables 2 and 3), whereas the remaining 35 genes showed discordant directions of expression change between the two cell lines. Among the concordantly upregulated DEGs, ITGA1 and CARMIL2 were identified, whereas BRWD3 was identified among the concordantly downregulated DEGs. GO analysis revealed enrichment of biological processes related to macromolecule biosynthesis (Table 4).

**Table 2:** Upregulated DEGs following Dsg3 knockdown under AD conditions.

UP-DEGs (AD)					
Gene ID	Gene Name	Gene Type	Entrez Gene ID	FoldChange (log2)	
				7LY	SAS
ENSG00000236782.7	AL391650.1	protein_coding	–	1.4800868	1.2126084
ENSG00000069702.11	TGFBR3	protein_coding	7049	1.2386185	2.2705289
ENSG00000116128.11	BCL9	protein_coding	607	1.4801397	1.3912814
ENSG00000287086.1	AC034193.1	lncRNA	–	1.0431977	1.3425518
ENSG00000240602.7	AADACP1	transcribed_unprocessed_pseudogene	201651	1.2263657	1.0052562
ENSG00000109689.17	STIM2	protein_coding	57620	1.0869309	1.0671142
ENSG00000218336.9	TENM3	protein_coding	55714	1.3742273	1.3722013
ENSG00000112972.15	HMGCS1	protein_coding	3157	2.1224584	1.1466484
ENSG00000213949.10	ITGA1	protein_coding	3672	1.0544204	2.0648003
ENSG00000164211.13	STARD4	protein_coding	134429	1.0350078	1.0987587
ENSG00000026950.17	BTN3A1	protein_coding	11119	1.1293342	1.7936067
ENSG00000135596.18	MICAL1	protein_coding	64780	1.1181248	1.3105433
ENSG00000196569.13	LAMA2	protein_coding	3908	1.5883934	1.7646219
ENSG00000022567.10	SLC45A4	protein_coding	57210	1.2097085	1.424822
ENSG00000107331.17	ABCA2	protein_coding	20	1.0901055	1.0687861
ENSG00000111671.9	SPSB2	protein_coding	84727	1.3409098	1.5634439
ENSG00000123384.14	LRP1	protein_coding	4035	1.4299878	1.1281048
ENSG00000135454.14	B4GALNT1	protein_coding	2583	1.6346239	1.0906607
ENSG00000172379.21	ARNT2	protein_coding	9915	1.9719856	1.447113
ENSG00000127561.15	SYNGR3	protein_coding	9143	1.0075583	1.9980169
ENSG00000269937.1	AC093525.7	lncRNA	–	1.1470246	1.1276762
ENSG00000205746.9	AC126755.1	transcribed_unprocessed_pseudogene	–	1.382213	2.5869321
ENSG00000090905.19	TNRC6A	protein_coding	27327	1.2361946	1.0395747
ENSG00000260280.5	SLX1B-SULT1A4	lncRNA	100526831	1.0169068	1.6155878
ENSG00000197162.10	ZNF785	protein_coding	146540	1.0051538	1.4970063
ENSG00000159753.14	CARMIL2	protein_coding	146206	1.1672419	1.4740388
ENSG00000196646.12	ZNF136	protein_coding	7695	1.2222611	1.0205474
ENSG00000126461.15	SCAF1	protein_coding	58506	1.7864755	1.4974997
ENSG00000130584.12	ZBTB46	protein_coding	140685	1.0623152	1.3148733
ENSG00000099904.16	ZDHHC8	protein_coding	29801	1.0908343	1.0022501

**Table 3:** Downregulated DEGs following Dsg3 knockdown under AD conditions.

DN-DEGs (AD)					
Gene ID	Gene Name	Gene Type	Entrez Gene ID	FoldChange (log2)	
				7LY	SAS
ENSG00000196517.13	SLC6A9	protein_coding	6536	−1.22429561	−1.53955926
ENSG00000183023.18	SLC8A1	protein_coding	6546	−1.78659636	−1.87897051
ENSG00000172878.14	METAP1D	protein_coding	254042	−1.43485568	−1.0232576
ENSG00000164342.13	TLR3	protein_coding	7098	−1.58984472	−1.65429201
ENSG00000096093.16	EFHC1	protein_coding	114327	−1.14061717	−1.55889095
ENSG00000231527.7	FAM27C	lncRNA	105379444	−1.43595596	−1.94725147
ENSG00000187210.14	GCNT1	protein_coding	2650	−1.43658622	−1.44896933
ENSG00000178440.7	TIMM23B-AGAP6	protein_coding	–	−1.25858262	−1.52837897
ENSG00000175548.9	ALG10B	protein_coding	144245	−1.74075717	−1.55083618
ENSG00000170442.12	KRT86	protein_coding	3892	−1.40904451	−1.4631979
ENSG00000139631.18	CSAD	protein_coding	51380	−1.03329886	−1.80541842
ENSG00000198270.13	TMEM116	protein_coding	89894	−1.26685718	−1.2947547
ENSG00000140044.13	JDP2	protein_coding	122953	−1.0332932	−1.65670602
ENSG00000159217.10	IGF2 BP1	protein_coding	10642	−1.19984259	−1.12961701
ENSG00000224738.1	AC099850.1	lncRNA	–	−1.01927126	−1.22549299
ENSG00000266401.2	AP002478.1	lncRNA	105371967	−2.42844175	−1.06316029
ENSG00000105750.15	ZNF85	protein_coding	7639	−1.5076674	−1.36735051
ENSG00000169239.13	CA5B	protein_coding	11238	−1.36197626	−1.03349691
ENSG00000165288.11	BRWD3	protein_coding	254065	−1.4116309	−1.33514738

**Table 4:** Summary of the top 20 biological process (BP-FAT) in Gene ontology (GO) analysis of UP-DEGs under AD conditions.

GO BP-FAT (AD)				
GO ID	GO Term	Count	%	P Value
GO:0034645	cellular macromolecule biosynthetic process	30	41.1	0.0074
GO:2000112	regulation of cellular macromolecule biosynthetic process	28	38.4	0.0025
GO:0010556	regulation of macromolecule biosynthetic process	28	38.4	0.0028
GO:0010468	regulation of gene expression	28	38.4	0.0115
GO:0016070	RNA metabolic process	27	37.0	0.0246
GO:0051252	regulation of RNA metabolic process	26	35.6	0.0007
GO:0019219	regulation of nucleobase-containing compound metabolic process	26	35.6	0.0031
GO:0018130	heterocycle biosynthetic process	25	34.2	0.0077
GO:0019438	aromatic compound biosynthetic process	25	34.2	0.0080
GO:0006351	transcription, DNA-templated	24	32.9	0.0016
GO:0006355	regulation of transcription, DNA-templated	24	32.9	0.0019
GO:1903506	regulation of nucleic acid-templated transcription	24	32.9	0.0019
GO:2001141	regulation of RNA biosynthetic process	24	32.9	0.0020
GO:0097659	nucleic acid-templated transcription	24	32.9	0.0029
GO:0032774	RNA biosynthetic process	24	32.9	0.0032
GO:0034654	nucleobase-containing compound biosynthetic process	24	32.9	0.0126
GO:0006357	regulation of transcription from RNA polymerase II promoter	23	31.5	0.0001
GO:0006366	transcription from RNA polymerase II promoter	16	21.9	0.0059
GO:0065009	regulation of molecular function	15	20.5	0.0501
GO:0007399	nervous system development	14	19.2	0.0978

Under AID conditions, a total of 56 overlapping candidate DEGs were identified in 7LY and SAS cells. Among these, 18 genes were concordantly upregulated and 16 genes were concordantly downregulated in both cell lines (Fig 6B; Tables 5 and 6), whereas the remaining 22 genes showed discordant directions of expression change between the two cell lines. Among the concordantly upregulated DEGs, ZNF462 and NAIP were identified, whereas CPEB2 was identified among the concordantly downregulated DEGs. GO analysis demonstrated enrichment of biological processes related to gene expression and nucleic acid biosynthesis, whereas no significant enrichment of pathways associated with cell death, apoptotic processes, or cell cycle regulation was observed (Table 7).

**Table 5:** Upregulated DEGs following Dsg3 knockdown under AID conditions.

UP-DEGs (AID)					
Gene ID	Gene Name	Gene Type	Entrez Gene ID	FoldChange (log2)	
				7LY	SAS
ENSG00000169914.6	OTUD3	protein_coding	23252	1.189824559	1.067250241
ENSG00000222009.8	BTBD19	protein_coding	149478	1.555563724	2.182203331
ENSG00000185219.17	ZNF445	protein_coding	353274	1.649502753	1.130494857
ENSG00000247950.7	SEC24B-AS1	lncRNA	100533182	1.24213134	1.554312288
ENSG00000249437.8	NAIP	protein_coding	4671	1.817813901	1.561016805
ENSG00000131711.15	MAP1B	protein_coding	4131	1.48429673	1.206167492
ENSG00000225791.7	TRAM2-AS1	lncRNA	401264	1.087193405	1.936150972
ENSG00000112182.15	BACH2	protein_coding	60468	2.206263745	1.234234034
ENSG00000205583.13	STAG3L1	transcribed_unprocessed_pseudogene	–	1.240759252	1.266220064
ENSG00000197646.8	PDCD1LG2	protein_coding	80380	2.101283336	1.437117033
ENSG00000148143.13	ZNF462	protein_coding	58499	1.267094254	1.127347718
ENSG00000204149.12	AGAP6	protein_coding	414189	1.038168378	1.240746339
ENSG00000174099.12	MSRB3	protein_coding	253827	1.239407204	1.468709432
ENSG00000263244.2	AC087190.3	lncRNA	–	1.518401337	1.856805364
ENSG00000169592.15	INO80E	protein_coding	283899	1.071868257	1.5780193
ENSG00000185829.18	ARL17A	protein_coding	51326	1.106975749	1.772243638
ENSG00000196757.8	ZNF700	protein_coding	90592	1.264710275	1.029154414
ENSG00000121406.9	ZNF549	protein_coding	256051	1.263885805	1.811789893

**Table 6:** Downregulated DEGs following Dsg3 knockdown under AID conditions.

DN-DEGs (AID)					
Gene ID	Gene Name	Gene Type	Entrez Gene ID	FoldChange (log2)	
				7LY	SAS
ENSG00000286185.1	AC242842.3	protein_coding	–	−1.314510623	−1.693215391
ENSG00000198929.13	NOS1AP	protein_coding	9722	−1.431146976	−1.154400399
ENSG00000162997.15	PRORSD1P	transcribed_unitary_pseudogene	344405	−1.332600747	−2.225960894
ENSG00000275111.5	ZNF2	protein_coding	7549	−1.393663848	−1.126152599
ENSG00000128683.14	GAD1	protein_coding	2571	−1.144667058	−1.096529246
ENSG00000115425.14	PECR	protein_coding	55825	−1.427770989	−1.007428384
ENSG00000163945.18	UVSSA	protein_coding	57654	−1.105000884	−1.096283652
ENSG00000137449.16	CPEB2	protein_coding	132864	−1.107704976	−1.225634434
ENSG00000182700.5	IGIP	protein_coding	492311	−1.235868654	−1.223216583
ENSG00000166436.16	TRIM66	protein_coding	9866	−2.271463028	−1.128897733
ENSG00000215039.7	CD27-AS1	lncRNA	678655	−1.059231122	−1.220719845
ENSG00000074621.14	SLC24A1	protein_coding	9187	−1.012065066	−2.800105183
ENSG00000266074.9	BAHCC1	protein_coding	57597	−1.770201924	−1.570012159
ENSG00000072071.16	ADGRL1	protein_coding	22859	−1.086360285	−1.419697508
ENSG00000105227.16	PRX	protein_coding	57716	−1.255905571	−1
ENSG00000105497.8	ZNF175	protein_coding	7728	−2.271906376	−1.120361179

**Table 7:** Summary of the top 20 biological process (BP-FAT) in Gene ontology (GO) analysis of UP-DEGs under AID conditions.

GO BP-FAT (AID)				
GO ID	GO Term	Count	%	PValue
GO:0010468	regulation of gene expression	17	33.3	0.024
GO:0051252	regulation of RNA metabolic process	15	29.4	0.009
GO:0019219	regulation of nucleobase-containing compound metabolic process	15	29.4	0.022
GO:2000112	regulation of cellular macromolecule biosynthetic process	15	29.4	0.046
GO:0010556	regulation of macromolecule biosynthetic process	15	29.4	0.049
GO:0006351	transcription, DNA-templated	14	27.5	0.013
GO:0006355	regulation of transcription, DNA-templated	14	27.5	0.014
GO:1903506	regulation of nucleic acid-templated transcription	14	27.5	0.014
GO:2001141	regulation of RNA biosynthetic process	14	27.5	0.015
GO:0097659	nucleic acid-templated transcription	14	27.5	0.019
GO:0032774	RNA biosynthetic process	14	27.5	0.020
GO:0034654	nucleobase-containing compound biosynthetic process	14	27.5	0.045
GO:0018130	heterocycle biosynthetic process	14	27.5	0.052
GO:0019438	aromatic compound biosynthetic process	14	27.5	0.053
GO:0010604	positive regulation of macromolecule metabolic process	12	23.5	0.057
GO:0009893	positive regulation of metabolic process	12	23.5	0.096
GO:0006357	regulation of transcription from RNA polymerase II promoter	11	21.6	0.032
GO:0009605	response to external stimulus	10	19.6	0.071
GO:0033554	cellular response to stress	8	15.7	0.096
GO:0009628	response to abiotic stimulus	6	11.8	0.077

## 4. Discussion

In this study, we demonstrated that altered Dsg3 expression, influenced by the anchorage environment, affects OSCC cell growth. An adhesive culture lacking extracellular matrix components was defined as AD conditions, while a suspension culture with low-adhesion treatment was defined as AID conditions. Our findings suggest that Dsg3 exerts two opposing effects on OSCC cell growth under AD and AID conditions. Under AD conditions, we observed significantly higher proliferation and migration abilities in Dsg3-low cells (7P, 17P, 58P) compared to Dsg3-high cells (7LY, 17LY, 58LY). Previous studies have reported contradictory roles for Dsg3 in OSCC growth, with both low and high Dsg3 expression linked to cancer progression. Several studies have associated the loss of desmosomal function with tumor metastasis [11,17], and clinicopathological studies have shown that reduced Dsg3 expression correlates with tumor growth, invasion, and lymph node metastasis [10–12]. Xin et al. reported that Dsg3 is localized to the cell membrane in normal epithelium but becomes internalized in cancer nests. Moreover, Dsg3 expression decreases at the invasion front, where its reduction is associated with higher lymph node metastasis [13]. Conversely, other studies have shown that Dsg3 promotes OSCC progression through intercellular signaling pathways. For instance, Brown et al. reported that Dsg3 enhances invasion and migration by regulating c-Jun/activator protein 1 (AP-1) activity and protein kinase C (PKC)-mediated Ezrin phosphorylation^2^. Chen et al. demonstrated that Dsg3 facilitates HNSCC proliferation by recruiting plakoglobin, which activates TCF/LEF downstream targets such as c-myc, cyclin D1, and MMP-7 [3]. These conflicting findings suggest that scaffolding factors may influence the effects of Dsg3, prompting us to examine its role under AID conditions.

Under AID conditions, Dsg3 expression was markedly upregulated in OSCC cells. Comparative analysis revealed that Dsg3-high cells (7LY, 17P, 58LY) had significantly greater proliferation and colony-forming abilities than Dsg3-low cells (7P, 17LY, 58P). Notably, Dsg3-negative cells (7P) formed smaller and fewer colonies under AID conditions. These findings suggest that, unlike under AD conditions, Dsg3-high cells contribute more significantly to tumor progression under AID conditions. Anoikis, a form of apoptosis triggered by loss of cell-matrix adhesion, is typically avoided by cancer cells, enabling their survival and proliferation under AID conditions [6–8]. Cirillo et al. reported that Dsg3 plays a crucial role in cell compaction and aggregation under AID conditions, where anti-Dsg3 antibodies significantly reduced the cohesion strength of spheroids [18].

In MCA cultures, the steady-state level of Dsg3 mRNA in OSCC cells (HSC3) increases as the cells enter the exponential growth phase but decreases upon reaching the plateau phase. On the other hand, other adhesion molecules are not so tightly regulated. For example, Dsg2 and catenins simply increased, while E-cadherin remained unchanged. These findings suggest that the upregulation of Dsg3 mRNA during the proliferative phase of MCA formation may indicate a specific and critical role of Dsg3 in the development of MCAs [19]. Baron et al. demonstrated that Dsg3-deficient keratinocytes exhibited adhesion defects and impaired tumor growth in allograft assays [20]. These findings highlight Dsg3’s role as a key adhesion factor for OSCC cell aggregation and tumor growth under AID conditions.

We further investigated the effects of Dsg3 knockdown on cell proliferation under AD and AID conditions. Under AD conditions, Dsg3 knockdown significantly increased proliferation in most Dsg3-expressing cell lines. However, in 58P and 58LY, the proliferative changes following Dsg3 knockdown under AD conditions did not reach statistical significance. Based on our data, it is suggested that in TOSCa-58, the magnitude of the proliferative change induced by Dsg3 knockdown was smaller compared with the other cell lines and may have remained within the range of experimental variability. Furthermore, although it is possible that the degree of dependence on Dsg3 may vary among cell lines depending on their biological background, it is difficult to identify the cause based solely on the current dataset, and this remains a subject for future investigation. Taken together, these findings do not contradict our conclusion that reduced Dsg3 expression contributes to increased proliferation under anchorage-dependent conditions. This interpretation is consistent with studies reporting that Dsg3 knockdown activates p38 mitogen-activated protein kinase (p38MAPK), promoting migration in HaCaT cells [21]. In contrast, other studies reported that Dsg3 knockdown suppresses tumor growth by inhibiting TCF/LEF transcriptional activity [3] and AP-1 phosphorylation [2]. These conflicting results highlight the complexity of Dsg3’s role under AD conditions. Under AID conditions, we hypothesized that Dsg3 knockdown would reduce OSCC cell proliferation. However, no significant changes were observed. Cirillo et al. suggested that while Dsg3 is not essential for MCA formation, it stabilizes cell aggregates through compaction [18]. In our study, MCA formation persisted despite Dsg3 knockdown, resulting in unchanged proliferation under AID conditions. Taken together, these findings suggest that Dsg3 may contribute to the cohesion and compaction of cancer cell aggregates, but is not indispensable for cell growth under AID conditions.

In addition, Dsg3 knockdown significantly reduced the size of multicellular aggregates without affecting their circularity under AID conditions. This finding suggests that Dsg3 contributes to the compaction or cohesion of cell aggregates rather than determining their overall morphology. The unchanged circularity indicates that, despite reduced aggregate size, the global geometry of the aggregates was largely preserved, implying that Dsg3 is not a primary determinant of aggregate shape but rather influences the internal structural organization of the aggregates.

Interestingly, although no significant difference in circularity was observed, the variability in circularity appeared to be increased in Dsg3 knockdown cells, as reflected by a wider distribution and larger standard deviation. This may indicate that Dsg3 contributes to the uniformity and structural consistency of MCAs.

To gain insight into the molecular basis of these findings, we performed an exploratory RNA-seq analysis to identify Dsg3-associated differentially expressed genes under AD and AID conditions. Although the exploratory analysis of DEGs overlapping between the patient-derived and commercial cell lines was not sufficient to draw definitive conclusions, we present these findings because a substantial number of cancer-related genes were identified. Under AD conditions, ITGA1 and CARMIL2 were identified as candidate UP-DEGs. ITGA1 is an adhesion molecule involved in tumor growth, local invasion, and CTC survival [22,23], while CARMIL2 enhances pseudopodia formation and cell migration [24]. These findings suggest that reduced Dsg3 expression under AD conditions may enhance migration and proliferation through molecules like ITGA1 and CARMIL2. Under AD conditions, BRWD3 was also identified as a downregulated DEG. BRWD3 is required for the regulation of cell shape by regulating cell morphology and cytoskeleton [25]. Under AID conditions, ZNF462 was identified as an upregulated DEG, whereas CPEB2 was identified as a downregulated DEG. ZNF462 regulates cell division and differentiation during early embryogenesis [26], while CPEB2 is critical for cell cycle progression [27]. Notably, the candidate UP-DEGs included NAIP, a well-known anti-apoptotic gene, further indicating that apoptotic signaling was not activated. Other DEGs were primarily associated with transcriptional regulation, chromatin remodeling, or cytoskeletal organization, and did not directly involve proliferation or survival pathways. These findings, together with the unchanged proliferative phenotype, suggest that Dsg3 knockdown is not associated with transcriptional changes related to apoptosis or reduced cell viability under AID conditions. Although dedicated apoptosis assays, such as Annexin V staining, were not performed in the present study, this remains an important subject for future investigation. Originally, we hypothesized that Dsg3 knockdown would reduce proliferation under AID conditions; however, the absence of changes in proliferation, together with the lack of evidence for apoptosis induction, suggests that Dsg3 may not directly regulate survival or growth in AID environments. Instead, these findings are consistent with the possibility that Dsg3 contributes to maintaining intercellular adhesion and aggregate stability under AID conditions, rather than to proliferative signaling. Gene ontology (GO) analysis under AD conditions suggested enrichment of biological processes related to macromolecule biosynthesis, whereas GO analysis under AID conditions suggested enrichment of processes associated with gene expression and nucleic acid biosynthesis. In contrast, GO analysis revealed no significant enrichment of pathways related to cell death, apoptotic processes, or cell cycle regulation under AID conditions. These findings suggest that Dsg3 knockdown is associated with distinct transcriptional responses under AD and AID conditions. However, the biological significance of the enriched GO terms, including those related to nucleic acid biosynthesis, remains unclear, and the present data do not establish a direct mechanistic link between these transcriptional changes and the observed phenotypic alterations, such as the reduction in multicellular aggregate size. Further functional studies will be required to determine whether these transcriptional changes contribute to the distinct biological phenotypes observed under each anchorage condition. In addition, the present GO analysis was limited to the Biological Process (BP FAT) category and did not include Cellular Component, Molecular Function, or pathway enrichment analyses such as KEGG or Reactome. Therefore, additional bioinformatics analyses will be required to further clarify the molecular pathways associated with Dsg3 function, including those potentially involved in cell adhesion and epithelial–mesenchymal transition. Furthermore, because of the limited number of samples, candidate DEGs were identified primarily on the basis of fold change rather than FDR-adjusted statistical significance. Therefore, the RNA-seq and GO analyses presented in this study should be interpreted as exploratory and hypothesis-generating, and further validation using larger datasets and independent functional experiments will be required.

Recent transcriptomic studies have similarly demonstrated the utility of integrated RNA-seq and bioinformatics analyses for identifying metastasis-associated molecular signatures in various cancers. For example, transcriptomic analyses of lung squamous cell carcinoma identified candidate genes associated with lymphatic metastasis, including ZNF334 [28], whereas subsequent studies further validated metastasis-related biomarkers such as LINC00894, YEATS2-AS1, and SUGP2 [29]. Although the candidate genes identified differ from those in the present study, these reports support the usefulness of RNA-seq-based approaches for discovering molecular alterations associated with metastatic progression. Our findings are consistent with these previous studies and further support transcriptome-based analyses as a useful strategy for identifying Dsg3-associated molecular changes under different anchorage conditions.

Recent studies have highlighted the importance of desmosomal proteins in cancer cell aggregation under anchorage-independent conditions. Li et al. demonstrated that DSC2 and PKP1 promote circulating tumor cell cluster formation and enhance cell survival and metastatic capacity through PI3K/AKT/Bcl-2-associated signaling [30]. Similarly, Sakurai et al. reported that oral squamous cell carcinoma cells formed desmosome-containing aggregates under low-attachment conditions and exhibited increased expression of the desmosomal genes DSC3 and DSP [31]. These findings are consistent with our observation that Dsg3 knockdown significantly reduced multicellular aggregate size under AID conditions. However, unlike the survival-promoting effects reported for DSC2 and PKP1, Dsg3 knockdown did not significantly alter proliferation in our model, suggesting that individual desmosomal proteins may exert distinct but complementary functions in aggregate formation and anchorage-independent survival. In addition to these findings, other desmosomal proteins have also been implicated in tumorigenesis. For example, plakoglobin is thought to promote the proliferation of squamous cell carcinoma (SCC) cells by inducing the survival-promoting gene Bcl-2 and inhibiting apoptosis. In addition, overexpression of plakophilin-3 has been reported to enhance tumor cell proliferation and motility. Furthermore, it has been suggested that epithelial-mesenchymal transition (EMT) regulators such as Snail and Slug can disrupt desmosomes, whereas conversely, the reassembly of desmosomes may lead to the reversal of EMT [32]. Based on these reports, the investigation of desmosomal proteins other than Dsg3 should also be considered, which represents one of the limitations of the present study. In addition, a limitation of the present study is that the analyses were performed using only three pairs of patient-derived primary and metastatic OSCC cell lines. Although paired cell lines provide a strong framework for comparing biological differences under shared genomic backgrounds, the limited sample size restricts the generalizability of the present findings. Therefore, our conclusions should be interpreted cautiously, and further validation using a larger number of patient-derived cell lines and independent cohorts will be necessary to confirm the broader applicability of our observations. In conclusion, Dsg3 exhibits dual roles in OSCC, promoting or suppressing growth depending on the anchorage environment. These findings highlight the importance of the scaffold environment in shaping Dsg3’s paradoxical functions and suggest that its dual roles enable OSCC cells to adapt to both primary tumor and metastatic conditions. These findings may provide insights into the role of desmosomal adhesion in OSCC progression and suggest that Dsg3-mediated adhesion mechanisms could represent potential therapeutic targets in metastatic OSCC.
