# Supplementary material for: Two opposite effects of desmoglein 3 on the growth of oral squamous cell carcinoma between anchorage -dependent and -independent conditions
Source: PLoS One. 2026 Sep 25;21(9):e0358339. doi: 10.1371/journal.pone.0358339 (PMC13614555; doi:10.1371/journal.pone.0358339)
Supplement: Supplementary file 1 [file pone.0358339.s001.doc]

We sincerely thank the Editor and the reviewers for their careful evaluation of our manuscript and for their constructive and insightful comments. We have revised the manuscript accordingly, and we believe that the revisions have significantly improved the clarity and scientific rigor of our study.

Below, we provide a point-by-point response to each comment. All changes have been incorporated into the revised manuscript.

This study explores a fascinating and important scientific question: the functional paradox of Dsg3 in OSCC. Its core finding—that the role of Dsg3 is context-dependent and anchoring—is highly innovative and potentially of significant biological relevance. The use of paired primary/metastatic cell lines is a major strength of the experimental design. However, the research is still at a preliminary exploratory stage, and the evidence supporting the main conclusions has several critical weaknesses, with the mechanistic investigation appearing particularly initial.

1. Although the use of paired cell lines is a notable advantage of this study, the conclusions are drawn from only 3 pairs of patient cell lines. The small sample size may limit the generalizability of the findings. Please explicitly acknowledge this limitation in the Discussion section.

We appreciate this insightful comment. We agree that the number of paired primary/metastatic cell lines is limited, and this may restrict the generalizability of our conclusions. This point has now been explicitly acknowledged in the revised Discussion. We also emphasize in the revision that, although the use of paired cell lines provides a unique advantage for evaluating biological differences between primary and metastatic lesions under identical genetic backgrounds, further validation using a larger panel of patient-derived cell lines will be required to establish the universality of our findings.

**Revision in the manuscript (Discussion section):**
“A limitation of the present study is that the analyses were performed using only three pairs of patient-derived primary and metastatic OSCC cell lines. Although paired cell lines provide a strong framework for comparing biological differences under shared genomic backgrounds, further studies using a larger number of patient-derived samples will be necessary to validate the generalizability of our conclusions.” (Page 25, Lines 426– Page 26, 431)

2. The authors found that knocking down Dsg3 under AID conditions had no effect on proliferation and speculated that its role lies in "stabilizing aggregates" rather than driving proliferation. However, direct evidence is lacking, and the logic is insufficient. Please provide data on cell viability and apoptosis. If knocking down Dsg3 under AID conditions leads to increased cell death rather than reduced proliferation, it would better support the argument.

We sincerely appreciate this important and insightful comment. The reviewer raised a critical concern regarding whether the unchanged proliferation observed after Dsg3 knockdown under AID conditions may reflect a balance between reduced proliferation and increased cell death, rather than a true lack of effect on cell growth.

To address this point, we re-evaluated our data with a focus on cell viability and apoptosis. Although no reduction in proliferation was observed in the WST-1 assay under AID conditions, it is important to note that WST-1 reflects both cell proliferation and viability. Therefore, the absence of a decrease in WST-1 signal suggests that Dsg3 knockdown does not reduce cell viability.

This interpretation is further supported by our RNA-seq analysis. Gene Ontology (GO) analysis did not reveal enrichment of pathways related to “cell death,” “apoptotic process,” or “cell cycle regulation,” and no major apoptosis-associated genes were identified among the differentially expressed genes (DEGs). In addition, NAIP, a well-known anti-apoptotic factor, was upregulated, further supporting the absence of apoptotic signaling.

Taken together, these findings strongly suggest that the unchanged proliferation observed after Dsg3 knockdown under AID conditions is not attributable to increased cell death or reduced viability.

To further address the reviewer’s concern, we additionally performed analyses focusing on the structural characteristics of multicellular aggregates. Specifically, we quantified aggregate size and circularity following Dsg3 knockdown. As a result, aggregate size was significantly reduced, whereas circularity remained largely unchanged, with increased variability.

These findings suggest that Dsg3 contributes to the cohesion and compaction of multicellular aggregates rather than regulating cell proliferation or survival.

These revisions have been incorporated into the Discussion and Results sections of the revised manuscript.

3. Please discuss the possible reasons for the non-significant changes in proliferation observed in Figure 4B for 58P and 58LY.

Thank you very much for your valuable comment. In this study, Dsg3 knockdown under AD conditions showed an increasing trend in proliferation in many cell lines, whereas no statistically significant change was observed in 58P and 58LY. We consider the following points as possible explanations.

First, in 58P/58LY, the magnitude of the change in proliferative ability after Dsg3 knockdown was smaller than in the other cell lines and may have remained within the range of intra-experimental variability. In fact, the proliferation tended to increase upon Dsg3 knockdown, and the direction of change was not markedly inconsistent with the other cell lines; however, the change was not large enough to reach statistical significance.

In addition, the overall results of this study suggest that the degree of dependence on Dsg3 varies among cell lines depending on their biological background. However, based on the current dataset alone, it is difficult to identify the underlying cause, and we consider this an important issue for future investigation.

Therefore, we interpret the absence of statistically significant proliferation changes in 58P and 58LY as indicating that the function of Dsg3 may depend on the cell line–specific molecular background or signaling network, which is consistent with our conclusion that “the role of Dsg3 is context-dependent.” This point has been added to the revised Discussion.

4. Figure 3 shows that Dsg3 expression is higher in 17P than in 17LY, and the colony formation assay also indicates that 17P cells have stronger proliferative ability than 17LY. This appears inconsistent with the description: "Dsg3-high cells (7LY, 17LY, 58LY) exhibited significantly greater proliferation than their Dsg3-low counterparts (7P, 17P, 58P) (Figs 3A, B)." Please explain this discrepancy.

Thank you very much for pointing this out. This was due to a typographical error in the manuscript. The correct description is as follows:

“Dsg3-high cells (7LY, 17P, 58LY) exhibited significantly greater proliferation than their Dsg3-low counterparts (7P, 17LY, 58P) (Figs 3A, B).”

As shown in Figure 3, 17P exhibits higher Dsg3 expression and higher colony-forming ability compared with 17LY, and therefore the correct grouping is as written above. This correction does not affect the interpretation of the data or the overall conclusion of the study. The revised manuscript has been updated accordingly.
