# Supplementary material for: Two opposite effects of desmoglein 3 on the growth of oral squamous cell carcinoma between anchorage -dependent and -independent conditions
Source: PLoS One. 2026 Sep 25;21(9):e0358339. doi: 10.1371/journal.pone.0358339 (PMC13614555; doi:10.1371/journal.pone.0358339)
Supplement: Supplementary file 2 [file pone.0358339.s002.doc]

This manuscript investigates the context-dependent role of desmoglein 3 (Dsg3) in oral squamous cell carcinoma (OSCC) under anchorage-dependent (AD) and anchorage-independent (AID) conditions. The topic is interesting because contradictory roles of Dsg3 have been reported previously, and the comparison between AD and AID conditions provides a novel perspective. The use of paired primary tumor and metastatic cell lines is also a strength of the study.

**Major comments**

1. **Limited number of patient-derived cell lines.** Although the paired primary/metastatic cell lines represent an important strength, only three patient-derived pairs were included. The authors acknowledge this limitation in the revised Discussion, but the restricted sample size still limits the generalizability of the findings.
2. **Functional interpretation of GO analysis.** GO enrichment identifies biological processes associated with Dsg3 knockdown but does not establish causal mechanisms. Accordingly, statements implying direct regulation of proliferation or apoptosis should be softened unless supported by additional functional experiments.

**Minor comments**

1. The manuscript would benefit from additional discussion comparing the present findings with recent studies on desmosomal proteins under anchorage-independent growth conditions.
2. The Discussion could better distinguish between observations directly supported by experimental data and hypotheses proposed by the authors.
3. Statistical methods should clearly indicate whether normality was assessed before selecting Student's *t*-test or Mann–Whitney *U* test.
4. The authors may consider discussing additional recent transcriptomic studies that identified metastasis-associated genes through integrated bioinformatics analyses (e.g., studies on ZNF334, LINC00894/YEATS2-AS1/SUGP2, and lymphatic metastasis-associated genes in lung cancer and other Genes whose expressions in the primary lung squamous cell carcinoma), as these studies provide complementary evidence supporting the application of RNA-seq-based approaches for discovering metastasis-related molecular signatures.
5. The English language is generally good, although minor grammatical editing would improve readability.
