# Supplementary material for: Two opposite effects of desmoglein 3 on the growth of oral squamous cell carcinoma between anchorage -dependent and -independent conditions
Source: PLoS One. 2026 Sep 25;21(9):e0358339. doi: 10.1371/journal.pone.0358339 (PMC13614555; doi:10.1371/journal.pone.0358339)
Supplement: Supplementary file 3 [file pone.0358339.s003.doc]

**Major concerns:**

1. The threshold for DEGs (log₂ fold change ≥1 or ≤-1) lacks statistical stringency (e.g., no adjustment for multiple testing via FDR/Benjamini-Hochberg correction).

We thank the reviewer for this important comment. We agree that FDR-adjusted statistical thresholds provide greater stringency for DEG identification. Statistical analysis was initially performed; however, no genes met the FDR-adjusted significance threshold. Therefore, because the purpose of this analysis was exploratory, candidate genes were selected on the basis of the magnitude of expression change using a log₂ fold change threshold of ≥1 or ≤−1. We have clarified this analytical approach and its limitations in the revised Methods and Discussion. (Page 10 Lines 163– Page 11 171, Page 28 Lines 454–459)

2. GO analysis is limited to “biological processes” (BP FAT) without exploring cellular components, molecular functions, or pathway-level insights (e.g., KEGG, Reactome). Critical pathways like cell adhesion, EMT, are not rigorously evaluated.

We thank the reviewer for this valuable comment. We agree that complementary analyses, including Gene Ontology Cellular Component (CC), Molecular Function (MF), and pathway enrichment analyses such as KEGG and Reactome, would provide additional biological insight into the molecular functions associated with Dsg3. In the present study, GO analysis was intentionally limited to the Biological Process (BP FAT) category to obtain an overview of the biological processes associated with Dsg3 knockdown. We have acknowledged this limitation in the revised Discussion and emphasized that more comprehensive pathway analyses will be an important subject for future studies. (Page 27 Lines 445– Page 28 Line 450)

3. The study claims DEGs under AID conditions are linked to “nucleic acid biosynthesis” but provides no evidence for how this connects to Dsg3’s role. Mechanistic links between enriched GO terms and observed phenotypes (e.g., aggregate size) are missing.

We thank the reviewer for this important comment. We agree that GO enrichment analysis alone does not establish mechanistic links between enriched biological processes and the observed phenotypes. We have therefore revised the Discussion to clarify that the GO analysis provides supportive and exploratory information regarding transcriptional changes associated with Dsg3 knockdown. In particular, we emphasize that the enrichment of processes related to nucleic acid biosynthesis does not demonstrate a direct mechanistic relationship with the reduction in multicellular aggregate size, and that further functional studies are required to clarify this relationship. (Page 26 Line 430 – Page27 Lines 445)

4. Since DEGs and GO analyses were important to explain the function of Dsg3, these related results should put in formal text, but not as the supplementary file.

We thank the reviewer for this valuable comment. We agree that the RNA-seq findings are important for interpreting the role of Dsg3 in OSCC. Accordingly, we have transferred the DEG and GO analysis figures and tables to the main manuscript and revised the Results section accordingly. We also moved the corresponding Venn diagrams and DEG/GO summary tables to the main manuscript and revised the Results section to present the key findings more clearly. (Page 18 Lines 291– Page 20 Line 325)

This manuscript investigates the context-dependent role of desmoglein 3 (Dsg3) in oral squamous cell carcinoma (OSCC) under anchorage-dependent (AD) and anchorage-independent (AID) conditions. The topic is interesting because contradictory roles of Dsg3 have been reported previously, and the comparison between AD and AID conditions provides a novel perspective. The use of paired primary tumor and metastatic cell lines is also a strength of the study.

**Major comments**

1. **Limited number of patient-derived cell lines.** Although the paired primary/metastatic cell lines represent an important strength, only three patient-derived pairs were included. The authors acknowledge this limitation in the revised Discussion, but the restricted sample size still limits the generalizability of the findings.

We sincerely thank the reviewer for this important comment. We agree that the limited number of patient-derived paired cell lines represents a limitation of the present study and may restrict the generalizability of our findings. We have revised the Discussion to further emphasize that our conclusions should be interpreted as evidence obtained from a limited number of paired cell lines and that validation using a larger cohort of patient-derived models will be necessary in future studies. (Page 27 Lines 450 – Page 28 Line 455)

2. **Functional interpretation of GO analysis.** GO enrichment identifies biological processes associated with Dsg3 knockdown but does not establish causal mechanisms. Accordingly, statements implying direct regulation of proliferation or apoptosis should be softened unless supported by additional functional experiments.

We sincerely thank the reviewer for this valuable comment. We agree that Gene Ontology (GO) enrichment analysis identifies biological processes associated with Dsg3 knockdown but does not establish causal mechanisms. Accordingly, we have carefully revised the Discussion to avoid statements implying direct regulation of proliferation or apoptosis by Dsg3 based solely on GO analysis. Throughout the revised manuscript, conclusions derived from GO analysis have been rephrased using more cautious expressions such as "suggest," "may contribute," and "are consistent with." We also clearly distinguish observations supported directly by our experimental data from hypotheses based on transcriptomic analyses. (Page 26, Lines 423–425)

**Minor comments**

1. The manuscript would benefit from additional discussion comparing the present findings with recent studies on desmosomal proteins under anchorage-independent growth conditions.

We thank the reviewer for this valuable comment. We have expanded the Discussion to compare our findings with recent studies investigating desmosomal proteins under anchorage-independent conditions. Li et al. demonstrated that the desmosomal proteins DSC2 and PKP1 promote circulating tumor cell cluster formation and enhance cell survival and metastatic capacity. In addition, Sakurai et al. reported that oral cancer cells formed desmosome-containing aggregates under low-attachment conditions, accompanied by increased expression of the desmosomal genes DSC3 and DSP. We have discussed these findings in relation to our observation that Dsg3 knockdown reduced multicellular aggregate area without significantly affecting proliferation under anchorage-independent conditions. (Page 28 Line 467 – Page 29 Lines 479)

2. The Discussion could better distinguish between observations directly supported by experimental data and hypotheses proposed by the authors.

We thank the reviewer for this valuable comment. We agree that it is important to clearly distinguish between observations directly supported by our experimental data and hypotheses proposed to explain the underlying mechanisms. Accordingly, we have revised the Discussion throughout the manuscript to clearly separate these two aspects. Statements directly supported by our experimental findings are now presented as observations, whereas mechanistic interpretations are explicitly described as hypotheses using cautious expressions such as "suggest," "may," "it is possible," and "are consistent with the possibility." In addition, we have explicitly stated where further functional studies are required to verify the proposed mechanisms. We believe these revisions improve the clarity of the Discussion and avoid overinterpretation of the present findings.

3. Statistical methods should clearly indicate whether normality was assessed before selecting Student's *t*-test or Mann–Whitney *U* test.

We thank the reviewer for this valuable comment. We agree that the original manuscript did not sufficiently describe the criteria for selecting the statistical tests. We have revised the Statistical Analysis section to clarify that formal normality testing was not performed because of the limited number of observations and that statistical tests were selected according to the characteristics of each dataset. In our analyses, the Mann–Whitney U test was applied only to the pooled comparison shown in Fig. 2B, whereas Student's t-test was used for the remaining comparisons. (Page 11, Lines 174–180)

4. The authors may consider discussing additional recent transcriptomic studies that identified metastasis-associated genes through integrated bioinformatics analyses (e.g., studies on ZNF334, LINC00894/YEATS2-AS1/SUGP2, and lymphatic metastasis-associated genes in lung cancer and other Genes whose expressions in the primary lung squamous cell carcinoma), as these studies provide complementary evidence supporting the application of RNA-seq-based approaches for discovering metastasis-related molecular signatures.

We thank the reviewer for this valuable suggestion. We have revised the Discussion to include recent transcriptomic studies that identified metastasis-associated genes, including ZNF334, LINC00894, YEATS2-AS1, and SUGP2, through integrated RNA-seq and bioinformatics analyses. We also discuss how these studies complement our findings by supporting the utility of RNA-seq-based approaches for identifying metastasis-associated molecular signatures, although the specific candidate genes identified differ from those in the present study. Accordingly, we believe these additions provide broader context for our transcriptomic analysis and strengthen the Discussion. (Page 28 Lines 456–466)

5. The English language is generally good, although minor grammatical editing would improve readability.

We thank the reviewer for this helpful comment. We have carefully reviewed the manuscript and made minor grammatical and stylistic revisions throughout the text to improve readability and clarity.
