# Supplementary material for: Two opposite effects of desmoglein 3 on the growth of oral squamous cell carcinoma between anchorage -dependent and -independent conditions
Source: PLoS One. 2026 Sep 25;21(9):e0358339. doi: 10.1371/journal.pone.0358339 (PMC13614555; doi:10.1371/journal.pone.0358339)
Supplement: Peer review history

# Peer review: Decision Letter 0

**DOI:** 10.1371/journal.pone.0358339.r001

**Authors:** Melvin Anyasi Ambele

27 Oct 2025

Dear Dr. Minabe,

Thank you for submitting your manuscript to PLOS ONE. After careful consideration, we feel that it has merit but does not fully meet PLOS ONE’s publication criteria as it currently stands. Therefore, we invite you to submit a revised version of the manuscript that addresses the points raised during the review process.

Please submit your revised manuscript by Dec 07 2025 11:59PM. If you will need more time than this to complete your revisions, please reply to this message or contact the journal office at plosone@plos.org. When you’re ready to submit your revision, log on to https://www.editorialmanager.com/pone/ and select the 'Submissions Needing Revision' folder to locate your manuscript file.

Please include the following items when submitting your revised manuscript:

- A rebuttal letter that responds to each point raised by the academic editor and reviewer(s). You should upload this letter as a separate file labeled 'Response to Reviewers'.
- A marked-up copy of your manuscript that highlights changes made to the original version. You should upload this as a separate file labeled 'Revised Manuscript with Track Changes'.
- An unmarked version of your revised paper without tracked changes. You should upload this as a separate file labeled 'Manuscript'.

If you would like to make changes to your financial disclosure, please include your updated statement in your cover letter. Guidelines for resubmitting your figure files are available below the reviewer comments at the end of this letter.

If applicable, we recommend that you deposit your laboratory protocols in protocols.io to enhance the reproducibility of your results. Protocols.io assigns your protocol its own identifier (DOI) so that it can be cited independently in the future. For instructions see: https://journals.plos.org/plosone/s/submission-guidelines#loc-laboratory-protocols. Additionally, PLOS ONE offers an option for publishing peer-reviewed Lab Protocol articles, which describe protocols hosted on protocols.io. Read more information on sharing protocols at https://plos.org/protocols?utm_medium=editorial-email&utm_source=authorletters&utm_campaign=protocols.

We look forward to receiving your revised manuscript.

Kind regards,

Melvin Anyasi Ambele, Ph.D

Academic Editor

PLOS ONE

Journal Requirements:

When submitting your revision, we need you to address these additional requirements.

1. Please ensure that your manuscript meets PLOS ONE's style requirements, including those for file naming. The PLOS ONE style templates can be found at

https://journals.plos.org/plosone/s/file?id=wjVg/PLOSOne_formatting_sample_main_body.pdf and

https://journals.plos.org/plosone/s/file?id=ba62/PLOSOne_formatting_sample_title_authors_affiliations.pdf

2. PLOS requires an ORCID iD for the corresponding author in Editorial Manager on papers submitted after December 6th, 2016. Please ensure that you have an ORCID iD and that it is validated in Editorial Manager. To do this, go to ‘Update my Information’ (in the upper left-hand corner of the main menu), and click on the Fetch/Validate link next to the ORCID field. This will take you to the ORCID site and allow you to create a new iD or authenticate a pre-existing iD in Editorial Manager.

3. We note that you have included the phrase “data not shown” in your manuscript. Unfortunately, this does not meet our data sharing requirements. PLOS does not permit references to inaccessible data. We require that authors provide all relevant data within the paper, Supporting Information files, or in an acceptable, public repository. Please add a citation to support this phrase or upload the data that corresponds with these findings to a stable repository (such as Figshare or Dryad) and provide and URLs, DOIs, or accession numbers that may be used to access these data. Or, if the data are not a core part of the research being presented in your study, we ask that you remove the phrase that refers to these data.

4. If the reviewer comments include a recommendation to cite specific previously published works, please review and evaluate these publications to determine whether they are relevant and should be cited. There is no requirement to cite these works unless the editor has indicated otherwise.

[Note: HTML markup is below. Please do not edit.]

Reviewers' comments:

Reviewer's Responses to Questions

**Comments to the Author**

1. Is the manuscript technically sound, and do the data support the conclusions?

Reviewer #1: Yes

Reviewer #2: Partly

**********

2. Has the statistical analysis been performed appropriately and rigorously?

Reviewer #1: Yes

Reviewer #2: Yes

**********

3. Have the authors made all data underlying the findings in their manuscript fully available?

The PLOS Data policy

Reviewer #1: Yes

Reviewer #2: Yes

**********

4. Is the manuscript presented in an intelligible fashion and written in standard English?

Reviewer #1: Yes

Reviewer #2: Yes

**********

Reviewer #1: This study explores a fascinating and important scientific question: the functional paradox of Dsg3 in OSCC. Its core finding—that the role of Dsg3 is context-dependent and anchoring—is highly innovative and potentially of significant biological relevance. The use of paired primary/metastatic cell lines is a major strength of the experimental design. However, the research is still at a preliminary exploratory stage, and the evidence supporting the main conclusions has several critical weaknesses, with the mechanistic investigation appearing particularly initial.

1. Although the use of paired cell lines is a notable advantage of this study, the conclusions are drawn from only 3 pairs of patient cell lines. The small sample size may limit the generalizability of the findings. Please explicitly acknowledge this limitation in the Discussion section.

2. The authors found that knocking down Dsg3 under AID conditions had no effect on proliferation and speculated that its role lies in "stabilizing aggregates" rather than driving proliferation. However, direct evidence is lacking, and the logic is insufficient. Please provide data on cell viability and apoptosis. If knocking down Dsg3 under AID conditions leads to increased cell death rather than reduced proliferation, it would better support the argument.

3. Please discuss the possible reasons for the non-significant changes in proliferation observed in Figure 4B for 58P and 58LY.

4. Figure 3 shows that Dsg3 expression is higher in 17P than in 17LY, and the colony formation assay also indicates that 17P cells have stronger proliferative ability than 17LY. This appears inconsistent with the description: "Dsg3-high cells (7LY, 17LY, 58LY) exhibited significantly greater proliferation than their Dsg3-low counterparts (7P, 17P, 58P) (Figs 3A, B)." Please explain this discrepancy.

Reviewer #2: Two opposite effects of desmoglein 3 on the growth of oral squamous cell carcinoma between anchorage -dependent and -independent conditions

A brief summary

This study examines the role of Dsg3 in OSCC growth under anchorage dependent (AD) and anchorage-independent (AID) conditions. The authors demonstrated that altered Dsg3 expression, influenced by the anchorage 262 environment, affects OSCC cell growth and they suggested that Dsg3 exerts two opposing effects on OSCC cell growth under AD and AID conditions.

Specific comments

1.In the abstract section, it is better that the authors give results using statistical parameters.

2.In the abstract section, it is better that the authors give conclusion in the end of that.

3. In the methods section, the authors should be explaining more details about the controls that they used in this study including positive and negative controls.

4. In the methods section, the authors should be explaining more details statistical methods.

5. In the results section, the authors should be writing a caption for the image with more explanation. Also, indicate on the graph what the horizontal and vertical axes are.

6. In the discussion section, the authors should be giving some practical suggestions.

**********

what does this mean?). If published, this will include your full peer review and any attached files.

If you choose “no”, your identity will remain anonymous but your review may still be made public.

**Do you want your identity to be public for this peer review?**  For information about this choice, including consent withdrawal, please see our Privacy Policy

Reviewer #1: No

Reviewer #2: **Yes:**  No

**********

[NOTE: If reviewer comments were submitted as an attachment file, they will be attached to this email and accessible via the submission site. Please log into your account, locate the manuscript record, and check for the action link "View Attachments". If this link does not appear, there are no attachment files.]

To ensure your figures meet our technical requirements, please review our figure guidelines: https://journals.plos.org/plosone/s/figures

You may also use PLOS’s free figure tool, NAAS, to help you prepare publication quality figures: https://journals.plos.org/plosone/s/figures#loc-tools-for-figure-preparation.

NAAS will assess whether your figures meet our technical requirements by comparing each figure against our figure specifications.

# Author comment: Author response to Decision Letter 1

**DOI:** 10.1371/journal.pone.0358339.r002

22 Apr 2026

We sincerely thank the reviewers for their valuable comments. We have carefully revised the manuscript accordingly, and detailed point-by-point responses have been provided in the uploaded “Response to Reviewers” document.

Attachment Submitted filename: Response to Reviewers.doc

# Peer review: Decision Letter 1

**DOI:** 10.1371/journal.pone.0358339.r003

**Authors:** Bingli Wu

21 Jul 2026

Dear Dr. Minabe,

Thank you for submitting your manuscript to PLOS ONE. After careful consideration, we feel that it has merit but does not fully meet PLOS ONE’s publication criteria as it currently stands. Therefore, we invite you to submit a revised version of the manuscript that addresses the points raised during the review process.

Please submit your revised manuscript by Sep 04 2026 11:59PM. If you will need more time than this to complete your revisions, please reply to this message or contact the journal office at plosone@plos.org. When you're ready to submit your revision, log on to https://www.editorialmanager.com/pone/ and select the 'Submissions Needing Revision' folder to locate your manuscript file.

- A letter that responds to each point raised by the academic editor and reviewer(s). You should upload this letter as a separate file labeled 'Response to Reviewers'.
- A marked-up copy of your manuscript that highlights changes made to the original version. You should upload this as a separate file labeled 'Revised Manuscript with Track Changes'.
- An unmarked version of your revised paper without tracked changes. You should upload this as a separate file labeled 'Manuscript'.

If you would like to make changes to your financial disclosure, please include your updated statement in your cover letter. Guidelines for resubmitting your figure files are available below the reviewer comments at the end of this letter.

If applicable, we recommend that you deposit your laboratory protocols in protocols.io to enhance the reproducibility of your results. Protocols.io assigns your protocol its own identifier (DOI) so that it can be cited independently in the future. For instructions see: https://journals.plos.org/plosone/s/submission-guidelines#loc-laboratory-protocols. Additionally, PLOS ONE offers an option for publishing peer-reviewed Lab Protocol articles, which describe protocols hosted on protocols.io. Read more information on sharing protocols at https://plos.org/protocols?utm_medium=editorial-email&utm_source=authorletters&utm_campaign=protocols.

As the corresponding author, your ORCID iD is verified in the submission system and will appear in the published article. PLOS supports the use of ORCID, and we encourage all coauthors to register for an ORCID iD and use it as well. Please encourage your coauthors to verify their ORCID iD within the submission system before final acceptance, as unverified ORCID iDs will not appear in the published article. *Only*  the individual author can complete the verification step; PLOS staff *cannot*  verify ORCID iDs on behalf of authors.

We look forward to receiving your revised manuscript.

Kind regards,

Bingli Wu

Academic Editor

PLOS One

Journal Requirements:

If the reviewer comments include a recommendation to cite specific previously published works, please review and evaluate these publications to determine whether they are relevant and should be cited. There is no requirement to cite these works unless the editor has indicated otherwise.

Additional Editor Comments (if provided):

This study investigates the context-dependent role of desmoglein 3 (Dsg3) in oral squamous cell carcinoma (OSCC) growth under anchorage-dependent (AD) and anchorage-independent (AID) conditions.

Major concerns:

1. The threshold for DEGs (log₂ fold change ≥1 or ≤-1) lacks statistical stringency (e.g., no adjustment for multiple testing via FDR/Benjamini-Hochberg correction).

2. GO analysis is limited to “biological processes” (BP FAT) without exploring cellular components, molecular functions, or pathway-level insights (e.g., KEGG, Reactome). Critical pathways like cell adhesion, EMT, are not rigorously evaluated.

3. The study claims DEGs under AID conditions are linked to “nucleic acid biosynthesis” but provides no evidence for how this connects to Dsg3’s role. Mechanistic links between enriched GO terms and observed phenotypes (e.g., aggregate size) are missing.

4. Since DEGs and GO analyses were important to explain the function of Dsg3, these related results should put in formal text, but not as the supplementary file.

[Note: HTML markup is below. Please do not edit.]

Reviewers' comments:

Reviewer's Responses to Questions

**Comments to the Author**

Reviewer #2: (No Response)

**********

2. Is the manuscript technically sound, and do the data support the conclusions?

Reviewer #2: Yes

**********

3. Has the statistical analysis been performed appropriately and rigorously?

Reviewer #2: Yes

**********

4. Have the authors made all data underlying the findings in their manuscript fully available?

The PLOS Data policy

Reviewer #2: Yes

**********

5. Is the manuscript presented in an intelligible fashion and written in standard English?

Reviewer #2: Yes

**********

Reviewer #2: This manuscript investigates the context-dependent role of desmoglein 3 (Dsg3) in oral squamous cell carcinoma (OSCC) under anchorage-dependent (AD) and anchorage-independent (AID) conditions. The topic is interesting because contradictory roles of Dsg3 have been reported previously, and the comparison between AD and AID conditions provides a novel perspective. The use of paired primary tumor and metastatic cell lines is also a strength of the study.

Major comments

1. Limited number of patient-derived cell lines. Although the paired primary/metastatic cell lines represent an important strength, only three patient-derived pairs were included. The authors acknowledge this limitation in the revised Discussion, but the restricted sample size still limits the generalizability of the findings.

2. Functional interpretation of GO analysis. GO enrichment identifies biological processes associated with Dsg3 knockdown but does not establish causal mechanisms. Accordingly, statements implying direct regulation of proliferation or apoptosis should be softened unless supported by additional functional experiments.

Minor comments

1. The manuscript would benefit from additional discussion comparing the present findings with recent studies on desmosomal proteins under anchorage-independent growth conditions.

2. The Discussion could better distinguish between observations directly supported by experimental data and hypotheses proposed by the authors.

3. Statistical methods should clearly indicate whether normality was assessed before selecting Student's t-test or Mann–Whitney U test.

4. The authors may consider discussing additional recent transcriptomic studies that identified metastasis-associated genes through integrated bioinformatics analyses (e.g., studies on ZNF334, LINC00894/YEATS2-AS1/SUGP2, and lymphatic metastasis-associated genes in lung cancer and other Genes whose expressions in the primary lung squamous cell carcinoma), as these studies provide complementary evidence supporting the application of RNA-seq-based approaches for discovering metastasis-related molecular signatures.

5. The English language is generally good, although minor grammatical editing would improve readability.

**********

what does this mean?). If published, this will include your full peer review and any attached files.

If you choose “no”, your identity will remain anonymous but your review may still be made public.

**Do you want your identity to be public for this peer review?**  For information about this choice, including consent withdrawal, please see our Privacy Policy

Reviewer #2: No

**********

[NOTE: If reviewer comments were submitted as an attachment file, they will be attached to this email and accessible via the submission site. Please log into your account, locate the manuscript record, and check for the action link "View Attachments". If this link does not appear, there are no attachment files.]

To ensure your figures meet our technical requirements, please review our figure guidelines: https://journals.plos.org/plosone/s/figures

You may also use PLOS’s free figure tool, NAAS, to help you prepare publication quality figures: https://journals.plos.org/plosone/s/figures#loc-tools-for-figure-preparation.

NAAS will assess whether your figures meet our technical requirements by comparing each figure against our figure specifications.

Attachment Submitted filename: Review for plos one.doc

# Author comment: Author response to Decision Letter 2

**DOI:** 10.1371/journal.pone.0358339.r004

17 Aug 2026

We sincerely thank the Academic Editor and Reviewer for their thoughtful and constructive comments. We have carefully addressed all comments and revised the manuscript accordingly. A detailed point-by-point response to each comment has been provided in the uploaded “Response to Reviewers” file. We believe that these revisions have substantially improved the clarity and rigor of the manuscript.

Attachment Submitted filename: Response_to_Reviewers_auresp_2.doc

# Editor report: Decision Letter 2

**DOI:** 10.1371/journal.pone.0358339.r005

**Authors:** Bingli Wu

1 Sep 2026

Two opposite effects of desmoglein 3 on the growth of oral squamous cell carcinoma between anchorage -dependent and -independent conditions

PONE-D-25-45451R2

Dear Dr. Minabe,

We’re pleased to inform you that your manuscript has been judged scientifically suitable for publication and will be formally accepted for publication once it meets all outstanding technical requirements.

Within one week, you’ll receive an e-mail detailing the required amendments. When these have been addressed, you’ll receive a formal acceptance letter and your manuscript will be scheduled for publication.

An invoice will be generated when your article is formally accepted. Please note, if your institution has a publishing partnership with PLOS and your article meets the relevant criteria, all or part of your publication costs will be covered. Please make sure your user information is up-to-date by logging into Editorial Manager at Editorial Manager® and clicking the ‘Update My Information' link at the top of the page. For questions related to billing, please contact billing support.

If your institution or institutions have a press office, please notify them about your upcoming paper to help maximize its impact. If they’ll be preparing press materials, please inform our press team as soon as possible -- no later than 48 hours after receiving the formal acceptance. Your manuscript will remain under strict press embargo until 2 pm Eastern Time on the date of publication. For more information, please contact onepress@plos.org.

Kind regards,

Bingli Wu

Academic Editor

PLOS One

Additional Editor Comments (optional):

Reviewers' comments:

# Editor report: Acceptance letter

**DOI:** 10.1371/journal.pone.0358339.r006

**Authors:** Bingli Wu

PONE-D-25-45451R2

PLOS One

Dear Dr. Minabe,

I'm pleased to inform you that your manuscript has been deemed suitable for publication in PLOS One. Congratulations! Your manuscript is now being handed over to our production team.

At this stage, our production department will prepare your paper for publication. This includes ensuring the following:

* All references, tables, and figures are properly cited

* All relevant supporting information is included in the manuscript submission,

* There are no issues that prevent the paper from being properly typeset

You will receive further instructions from the production team, including instructions on how to review your proof when it is ready. Please keep in mind that we are working through a large volume of accepted articles, so please give us a few days to review your paper and let you know the next and final steps.

Lastly, if your institution or institutions have a press office, please let them know about your upcoming paper now to help maximize its impact. If they'll be preparing press materials, please inform our press team within the next 48 hours. Your manuscript will remain under strict press embargo until 2 pm Eastern Time on the date of publication. For more information, please contact onepress@plos.org.

You will receive an invoice from PLOS for your publication fee after your manuscript has reached the completed accept phase. If you receive an email requesting payment before acceptance or for any other service, this may be a phishing scheme. Learn how to identify phishing emails and protect your accounts at https://explore.plos.org/phishing.

If we can help with anything else, please email us at customercare@plos.org.

Thank you for submitting your work to PLOS One and supporting open access.

Kind regards,

PLOS One Editorial Office Staff

on behalf of

Dr. Bingli Wu

Academic Editor

PLOS One
